# Therapeutic cell engineering: designing programmable synthetic genetic circuits in mammalian cells

**DOI:** 10.1007/s13238-021-00876-1

**Published:** 2021-09-29

**Authors:** Maysam Mansouri, Martin Fussenegger

**Affiliations:** 1 Department of Biosystems Science and Engineering, ETH Zurich, Mattenstrasse 26, 4058 Basel, Switzerland; 2 Faculty of Science, University of Basel, Mattenstrasse 26, 4058 Basel, Switzerland

**Keywords:** synthetic biology, cell-based therapy, cell engineering, therapeutic gene expression, controllable genetic circuits

## Abstract

Cell therapy approaches that employ engineered mammalian cells for on-demand production of therapeutic agents in the patient’s body are moving beyond proof-of-concept in translational medicine. The therapeutic cells can be customized to sense user-defined signals, process them, and respond in a programmable and predictable way. In this paper, we introduce the available tools and strategies employed to design therapeutic cells. Then, various approaches to control cell behaviors, including open-loop and closed-loop systems, are discussed. We also highlight therapeutic applications of engineered cells for early diagnosis and treatment of various diseases in the clinic and in experimental disease models. Finally, we consider emerging technologies such as digital devices and their potential for incorporation into future cell-based therapies.

## INTRODUCTION

Health-and-wellness programs emphasize prevention, early diagnosis and effective treatment options for disease management. Early detection can increase the efficacy of treatment, improve quality of life for patients, and also reduce the economic costs for both patients and health-care systems. However, current clinical interventions often happen only at late stages of disease due to a lack of early diagnosis (Schukur and Fussenegger, 2016). In most cases, patients consult with their physicians only when symptoms have already manifested, and as a result doctors have to prescribe drugs that target the disease and its symptoms instead of being able to prevent its onset. In addition, prescribed drugs often have to be administered following general rules, including strict timing intervals, and dosages have to be adjusted for the weight, age and gender of patients, which might not necessarily be appropriate for the actual physiological condition of individual patients (Kojima et al., 2020). Therefore, in contrast to the traditional “one-pill-fits-all” approach, next-generation medicine will require personalized strategies that allow for autonomous, early detection and provide options for immediate treatment by delivering the appropriate dosage of a therapeutic drug based on the stage of the disease.

Theranostics, a combination of therapeutics and diagnostics, employs implantable systems that are designed to automatically diagnose the patients’ disease status and to provide the correct treatment for the underlying medical condition (Kojima et al., 2016). There are two types of theranostic systems—electronic and biological. Both are equipped with sensitive sensors that are able to sense the levels of disease-related biomarkers and translate them to either a digital or biological output. The benchmark for electronic theranostic devices is the artificial pancreas, which can sense levels of glucose in the blood of diabetic patients and automatically inject the correct amount of insulin into the body through an integrated insulin pump (Brown et al., 2019). Such electronic devices have greatly improved the quality of life of patients, but their clinical application can be hampered by their complexity, and their use can also adversely influence the social life of patients (Slattery and Choudhary, 2017; Li et al., 2020). In addition, electronic devices have to be periodically refilled with therapeutic agents. In contrast, biological theranostic systems rely on implanted engineered cells, which can sense extracellular signals and respond according to a pre-defined therapeutic plan. Furthermore, the cells used in biological systems can work for extended periods of time if supplied with sufficient nutrients and energy.

**Figure 1:**
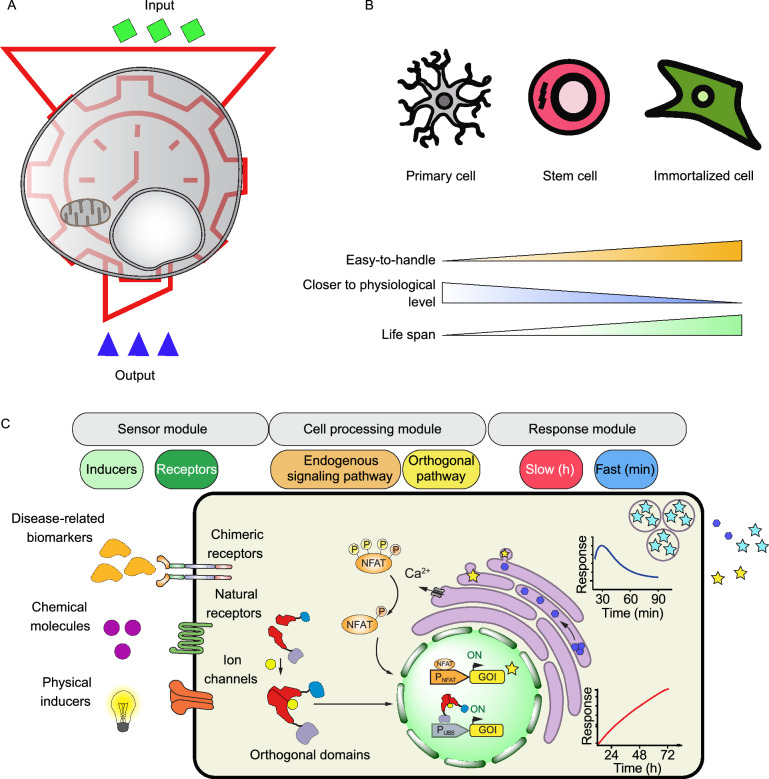
**Therapeutic cell engineering.** (A) Mammalian cells can be designed to receive a user-defined output, process it, and response in a programmable, controllable and predictable way. (B) Cell types used in synthetic biology-inspired cell-based therapies and their properties. (C) Major steps in engineering a therapeutic cell including design of a sensor module, a processing platform and a response plan. The sensor module consists of various inducers (disease-related biomarkers, chemical molecules and physical cues) and a range of receptors (native receptors, chimeric receptors, ion channels and switchable proteins) that can be stimulated by the inducers. Designer cells process the input signal through an endogenous signaling pathway (e.g., the calcium pathway depicted here) or an orthogonal route to produce a biological response. The response platform can be designed for slow (e.g., transcription of desired gene) or quick (e.g., fast release of pre-formed therapeutic proteins) action

Synthetic biology is the science of redesigning biological systems using genetic circuits to create useful entities with novel or improved capabilities (Xie and Fussenegger, 2015, 2018). Synthetic biology-inspired cell therapy strategies rely on genetically engineered cells, so-called designer cells, to sense a user-defined input signal, process it, and respond appropriately with a customizable therapeutic output (Fig. 1A) (Kitada et al., 2018). Usually, three types of engineered mammalian cells are used for therapeutic purposes: tissue-resident committed cells, stem cells and artificial cells. Naturally committed cells are specified for certain biological functions. Equipping these cells with novel synthetic genetic circuits can improve their functionalities through increased activity, specificity or efficiency. For example, immune cells that are engineered to express a synthetic receptor can combat disease that they were not able to efficiently handle before (Roybal and Lim, 2017). In contrast to committed cells, artificial cells are engineered cells that are not naturally involved in therapeutic responses in the field they are designed for. Instead, they are often malleable cells that are equipped with new functionalities. This group includes human embryonic kidney (HEK) cells, which have, for example, been engineered to mimic β cell function by sensing high levels of blood glucose and producing insulinogenic proteins in response (Xie et al., 2016b). Engineered stem cells form the third group of designer cells, which can contribute to clinical treatments either directly by production of therapeutic molecules or by facilitating regeneration of other therapeutic cells. For example, designer mesenchymal stem cells with inherent tropism to cancer cells can be used to deliver therapeutic agents to the tumor niche (Kojima et al., 2018). Alternatively, a synthetic lineage-control network in designer stem cells enables them to be differentiated into therapeutic cells (e.g., β cells) in a cost-effective, robust and reliable way (Saxena et al., 2016b, 2017).

In this review, we will introduce the synthetic toolbox that is available for therapeutic cell engineering. We will also look in more detail at self-sufficient engineered cells that regulate the production of a therapeutic agent in response to disease biomarkers autonomously (closed-loop) or in a controllable manner upon induction with user-provided chemical compounds or physical cues (open-loop). We also discuss emerging new technologies and their incorporation into next-generation cell-based therapies.

## SYNTHETIC BIOLOGY TOOLBOX FOR CELL ENGINEERING

Engineering of mammalian cells normally starts with the design and construction of a functional genetic circuit, which is subsequently delivered to appropriate primary cells, an immortalized cell line, or stem cells (Fig. 1B) (Khalil and Collins, 2010). Designer cell lines harboring alterations causing indefinite division are typically used in proof-of-concept studies. Otherwise, they would need to be protected from the response of the host’s immune system after transplantation. However, autologous primary cells, which retain many of the functions seen in vivo but which have a limited life span, as well as patient-derived stem cells, are very attractive for translational medicine and can potentially be transplanted without triggering an immune response (Maurisse et al., 2010; Eglen and Reisine, 2011). Compared to committed cells and stem cells, therapeutic artificial cells are easy to handle and are more compatible with scale-up procedures. Genetic materials are usually delivered to mammalian cells by chemical (e.g., cationic reagents like lipofectamine), physical (e.g., electroporation) or biological (e.g., viruses) approaches (Mansouri and Berger, 2018). The genetic components can be expressed transiently from exogenous plasmids or they can be integrated into the genome of engineered cells to ensure stable expression. Nowadays, targeted genome editing tools such as CRISPR/Cas9 can be harnessed to integrate the constructs into a defined locus in the host genome with high specificity and precision (Gaj et al., 2016).

In order to design a genetic circuit in mammalian cells, three main questions have to be considered carefully; what is the stimulus that will be applied to the cells, how should the cells process the stimulus, and how should the cells respond? (Fig. 1C). Inducers are often disease-related biomarkers, chemical molecules or other remote-controllable signals, all of which are discussed in detail in the following sections. To detect the stimulus, cells need to be equipped with an appropriate sensor system. Sensors are often a class of biological molecules that are able to detect and respond to target ligands with high specificity and sensitivity (Carpenter et al., 2018; Dixon et al., 2021). Typically, interaction between ligand and sensor causes a conformational change in the sensor’s structure, ultimately enabling the transfer of the input signal from the environment through the plasma membrane into the cytosol or nucleus of the cell. Plasma membrane receptors and (cytosolic) switchable proteins are the most common sensors that are used in synthetic biology (Scheller and Fussenegger, 2019). Native receptors have defined functions in their tissue of origin that can be harnessed to build engineered cells with similar functionality. For example, melanopsin is a blue-light-sensitive G protein-coupled receptor (GPCR), which is present in the eyes of animals and regulates their sleep (Mure et al., 2016), but expression of this receptor in non-retinal cells (e.g., HEK cells) can enable them to sense light as an input signal (Ye et al., 2011). Alternatively, chimeric receptors are engineered receptors comprised of domains from different proteins. These synthetic receptors can be designed to either detect a specific ligand for which there is no known natural receptor (Caliendo et al., 2019) or to enable a novel signaling pathway to reroute or replace the original. A good example of chimeric receptor engineering is the GEMS (generalized extracellular molecule sensor) platform, which makes it possible to establish synthetic receptors by adding a new ligand-binding domain (e.g., single-chain antibodies) on top of the native erythropoietin receptor (EpoR) to target a ligand of choice (Scheller et al., 2018). In addition, activation of the receptors can be rewired to various signaling pathways by replacing the intracellular signal transduction domain. Similar to plasma membrane receptors, switchable protein receptors are cytosolic receptors that can be customized for activation/inactivation upon interaction with their ligand (Weber and Fussenegger, 2004).

Activated sensors can either trigger endogenous signaling pathways or orthogonal routes (Xie and Fussenegger, 2018). In the other words, stimulated cells can activate an endogenous or orthogonal pathway to process an input signal and link it to a proper response. Plasma membrane receptors (native receptors, chimeric receptors and ion channels) often activate endogenous signaling pathways, triggering a rise of intracellular second messengers that leads to activation of transcription factors or changes in cellular metabolism, both of which can be rewired to a therapeutic effect (Kiel et al., 2010). In contrast, orthogonal systems are designed to minimize cross-talk between activated components and endogenous signaling pathways (McClune et al., 2019). These systems often rely on protein domains derived from other kingdoms of life. For example, synthetic orthogonal systems containing DNA-binding proteins from bacteria or yeast (e.g., TetR or Gal4) fused to viral transcriptional activators (e.g., VPR, VP16, VP64) are commonly used to control gene expression in mammalian cells (Yamada et al., 2020). In contrast to endogenous signaling pathways that can promote signal amplification through signal transduction cascades such as the MAPK pathway, orthogonal systems often work linearly without amplification. Therefore, orthogonal systems are needed in cases when either the integrity of endogenous signaling pathways is crucial for a specific function or when cross-talk with endogenous pathways would lead to undesired side-effects (Mukherjee et al., 2017).

The treatment of a disease requires a specific therapeutic response to be delivered by a cellular implant with the desired kinetics. Also, the dosage of the response needs to be designed according to the nature of the targeted disease. Different therapeutic responses can be achieved by using different production and secretion processes. Transcription of the desired (trans-)gene from the respective genomic locus (Su et al., 2020) or a synthetic expression unit (Xue et al., 2017), release of pre-formed therapeutics (Zhang and Tzanakakis, 2019), cellular differentiation (Saxena et al., 2016b) and cell migration (Park et al., 2014) are examples of inducible therapeutic responses that have been implemented in designer cells. Transcription-based therapeutic responses are generally slow (4–8 h after stimulation). Such prolonged kinetics is suitable for treating diseases that need lasting, long-term changes (e.g., expression of engineered receptors on immune cells to combat cancer). In contrast, there are other diseases where therapeutic proteins need to be administrated on a time scale of minutes or a few hours (e.g., insulin in type I diabetes) (Polonsky et al., 1988). Here, a quick response is essential. Therefore, designer cells should be engineered in a way that enables them to produce and store therapeutics internally in advance and release them in a burst upon stimulation. In this approach, transcription and translation are done in the uninduced state and cell stimulation only triggers trafficking and secretion of the therapeutic agent. Therapeutic proteins can be stored in and released from either synthetic vesicles (Krawczyk et al., 2020) or native organelles (e.g., ER or Golgi apparatus) (Rivera et al., 2000). The dosage of the response can be fine-tuned through factors such as ligand concentration, time of induction, and many others.

## CLOSED-LOOP-MEDIATED THERAPEUTIC GENETIC CIRCUITS

Molecular tools for cellular engineering are commonly used to control the activity or dosage of a therapeutic output (Lim and June, 2017). Currently, two types of control systems are available based on closed-loop and open-loop circuits that allow for precisely timed induction (Wang et al., 2018b). In a closed-loop system, a disease biomarker triggers a molecular feed-back loop in designer cells, leading to expression of a therapeutic protein that in turn causes the concentration of the biomarker to drop, down-regulating the system autonomously. We here divide closed-loop systems into two groups depending on whether the biomarker is soluble or anchored to the plasma membrane.

**Figure 2:**
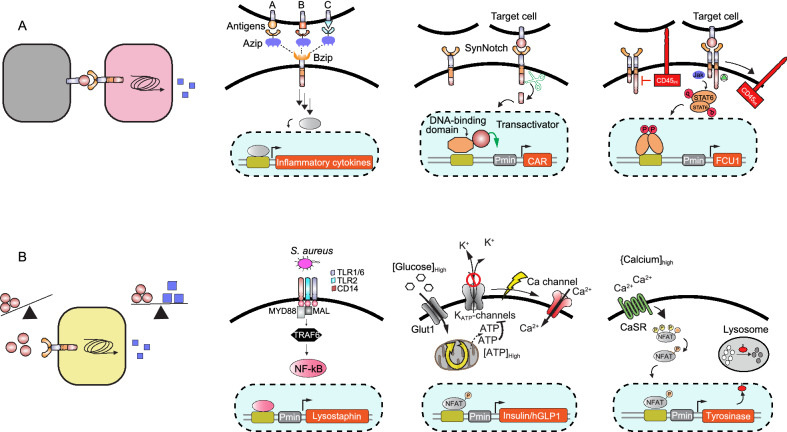
**Closed-loop-mediated cell-based therapies.** (A) Cell-to-cell mediated control of designer cells. This approach utilizes an antigen (biomarker) exposed on the surface of the targeted cell, and a physical interaction is needed for activation of the designer cell. SUPRA is a generalized CAR platform that enables a unique T cell to bind to different user-defined antigens and to trigger an endogenous signaling pathway upon activation. SynNotch relies on cleavage of a chimeric receptor after cell-cell interaction. The cleaved intracellular domain of synNotch translocates to the nucleus and initiates transcription of a desired therapeutic gene. Another cell-contact sensor is based on physical segregation of CD43ex-45int. In the absence of a target cell, CD43ex-45int suppresses an implemented JAK/STAT pathway. Once the designer cell binds to a targeted cell, CD43ex-45int is segregated from the cell–cell interface due to the physical force applied to the large extracellular domain of CD43ex-45int. Therefore, the JAK/STAT pathway will be activated and initiate expression of a therapeutic protein. (B) Metabolite-mediated closed-loop systems. A high level of soluble biomarker can stimulate designer cells to produce therapeutic agents to balance the level of the biomarker. In an immunomimetic cell, a TLR platform is used to activate the NFκB pathway and express an antibacterial peptide (lysostaphin) to treat MRSA. The β-cell-mimetic designer cell is designed to sense a high level of glucose in a diabetic model and to produce insulinogenic proteins (insulin and hGLP1) through expression of Ca_v_1.3 on HEK293 cells rewired to a synthetic expression unit. The biomedical tattoo utilizes a designer cell that enables early disease detection by sensing a high level of calcium, and produces tyrosinase upon activation. Tyrosinase mediates production of melanin, a black pigment, in the engineered designer cells

Membrane-bound biomarkers are normally presented on the surface of cells (e.g., cancer cells or cells infected with viruses) (Fig. 2A). In this case, physical cell-to-cell contact is required for ligand binding and activation of the designer cells. A well-known example of designer cells in this class is engineered T cells expressing a chimeric antigen receptor (CAR) on their membrane (Neelapu et al., 2018). Engineered T cells with a CAR targeting the cell-surface marker CD19 are approved by the FDA and are currently in use to treat different types of leukemia (e.g., Kymirah from Novartis) (June and Sadelain, 2018). A CAR is a chimeric receptor consisting of an scFv raised against the desired antigen (biomarker) fused to a transmembrane domain and a T cell-activating signaling domain on the intracellular side (Rafiq et al., 2020). Engineering T cells with CARs is an active field in T cell engineering and different strategies have been used to increase the efficacy of the engineered cells, including exchanging or expanding the intracellular signaling domains and customizing the genes that are activated upon induction (BiTE (Choi et al., 2019) and TRUCK (Chmielewski and Abken, 2015)). Researchers were also able to improve the specificity of the CARs through customizing the input and output signals. For example, a split, universal, and programmable (SUPRA) design of CARs enables a single T cell to bind to different user-defined antigens (Cho et al. 2018). This allows a universal designer T cell to regulate the stringency of the response according to the selected antigen. To do this, the extracellular domain of the CAR in the SUPRA system is split from the remaining part and both (extracellular and remaining part) are individually fused to a leucine zipper. Expression of the transmembrane-signaling fusion domain with the extracellular leucine zipper yields a universal acceptor CAR T cell. This acceptor cell can then be complemented by addition of different scFv-leucine zipper fusions to form a functional CAR. Another way to increase the specificity of CAR-T cells is to increase the number of biomarkers that can be recognized in parallel. This strategy can be used as a safety layer, and allows engineered CAR T cells to be activated only when either all specified biomarkers are available on a given target cell (AND-gated CAR T cells) or when only a specific set of biomarkers is available while another is not (A AND NOT B) (Fedorov et al., 2013; Kloss et al., 2013). In contrast to the success of CAR-T cell therapies for hematological malignancies, issues such as poor penetrability of T cells into solid tumors, the inhibitory tumor microenvironment (TME), and potential side effects have hampered applications to solid tumors. Therefore, modification of other types of cells with CAR has attracted great interest as an approach to overcome some of these hurdles. For example, macrophages transduced with CARs (CAR-M) can effectively contribute to antitumor responses and solid tumor eradication (Klichinsky et al., 2020). Other work capitalized on integrated sensing and activation proteins (iSNAP), which allow macrophages to ignore the self-defense signaling mechanism that tumor cells use to evade immune system responses. Specifically, iSNAP-expressing macrophages recognize the tumor cells’ escape signals as phagocytic signals (Sun et al., 2017). In addition, natural killer (NK) cells engineered with CAR (CAR-NK) show fewer adverse effects than T cell therapy, and are more amenable to off-the-shelf manufacturing (Albinger et al., 2021; Schmidt et al., 2021). Although CAR receptors are only functional in immune cells, cell-to-cell contact can also be modulated in other cell types (Fig. 2B). Synthetic Notch (synNotch) receptors, for example, are chimeric single-pass transmembrane receptors with a customized extracellular binding domain targeting a specific antigen (biomarker) that is fused to a transmembrane and intracellular signaling domain (Morsut et al., 2016). In contrast to CAR receptors, activated synNotch receptors trigger an orthogonal route to activate expression of a desired gene. In this case, mechanical force due to physical interaction between the T cell and its target exposes a hidden protease site in the transmembrane part of the synNotch receptor. This site can subsequently be cleaved by proteases, releasing a transcriptional activator that translocates to the nucleus and initiates transcription of desired genes. This system has been successfully employed for pattern formation during embryo development using engineered non-immune cells (Toda et al., 2018). Designer non-immune biosensor cells expressing synNotch receptors targeted to hepatitis B virus antigen could also produce antiviral proteins in response to virus-like particles (Matsunaga et al., 2020). In addition, expression of synNotch receptors has been used to increase or alter the specificity of CAR-T cells by on-target expression of the CAR receptor upon recognition of additional antigens on cancer cells (Roybal et al., 2016a) or by secretion of immune mediators to stimulate the immune system at the site of the tumor (Roybal et al., 2016b). To expand the targeting capacity of CAR and synNotch, a universal switchable strategy, called SNAP, has been developed to retarget engineered T cells to multiple antigens. In the SNAP-CAR/-synNotch system, the expression of a SNAP tag self-labeling enzyme on T cell membranes enables covalent interaction between the receptor and co-administered benzylguanine (BG)-conjugated antibodies (Lohmueller et al., 2020). Then, antibodies targeted to different antigens can activate the SNAP receptor and its downstream effector functions.

Another strategy based on membrane-anchored biomarkers in non-immune cells is co-expression of leaky chimeric IL4/13 receptors in combination with a signal-quenching phosphatase (CD43ex-45int) in HEK and MSC cells (Kojima et al., 2018). Chimeric IL4/13 receptors trigger a JAK-STAT pathway, which is hampered by the inhibitory effect of CD43ex-45int in the absence of membrane-bound ligand. When these engineered cells bind to a target cell, however, physical forces are thought to push CD43ex-45int away from the IL4/13 receptors, and this removes the suppressive effect on JAK-STAT signaling. JAK-STAT signaling is further re-routed to express a transgene of choice by a synthetic expression unit equipped with STAT-binding sites. This genetic circuit was successfully used in non-immune mesenchymal stem cells (MSC) to kill cancer cells by expression of a cell-penetrating fusion protein that converts an anti-cancer prodrug to a cytotoxic drug inside the target cell.

The second class of closed-loop circuits is activated by soluble biomarkers such as endogenous metabolites (Fig. 2C). A list of closed-loop systems that have been implemented in artificial cells and their sensor systems, as well as their therapeutic applications, is provided in Table 1. Rössger and colleagues have developed a designer cell that was able to sense levels of fatty acid in a mouse obesity model and produce pramlintide, a hormone that suppresses appetite, in response (Rössger et al., 2013a). They used a chimeric intracellular sensor system consisting of PPARα (peroxisome proliferator-activated receptor) fused to TtgR (phloretin-responsive repressor). Here, TtgR mediates binding of the PPARα-TtgR fusion protein to the operator sequence of a weak constitutive promoter, while PPARα controls gene expression through recruitment of different endogenous transcription modulators. In the absence of fatty acids, PPARα recruits an inhibitory complex to suppress transgene expression. Conversely, high levels of fatty acid trigger formation of an activation complex to turn the genetic circuit “ON”, leading to production of pramlintide. Accordingly, transgene expression can be regulated either by fatty acid in a self-sufficient closed-loop or by adding phloretin, the native ligand of TtgR, causing dissociation of inhibitory PPARα-TtgR from the synthetic promoter in an open-loop configuration. Pioneering work on closed-loop systems that are triggered by soluble disease-related biomarkers involved engineering HEK293 cells to sense high levels of glucose and respond with production of insulinogenic therapeutic protein to correct hyperglycemia in experimental mouse models of type 1 and type 2 diabetes (Xie et al., 2016b). These β-cell-mimetic designer cells were designed to express a voltage-gated calcium channel (Ca_v_1.3), which links glycolysis to Ca_v_1.3-mediated calcium influx. Co-transfection of a synthetic expression unit harboring the transgene (i.e., insulin and *GLP1*) under the control of a synthetic promoter including binding sites for endogenous transcription factors (i.e., NFAT) allows for expression of the desired therapeutic protein in response to hyperglycemia. Artificial beta cells (AβCs) are another example of engineered cells that mimic β-cell function by sensing high levels of glucose and secreting insulin through a vesicle-fusion mechanism. A high level of glucose triggers enzymatic oxidation and proton efflux in AβCs, creating a low pH environment in the cytoplasm. This low pH associated with hyperglycemia induces steric deshielding of peptides on inner small liposomal vesicles (ISVs) containing pre-formed and stored insulin, and these peptides form coiled-coil structures with peptides on outer large vesicles (OLVs), leading to release insulin from the ISVs (Chen et al., 2018). In addition to soluble metabolites involved in metabolic disease, Liu and colleagues developed an immunomimetic cell from non-immune cells, and showed that it is able to sense bacterial infection and produce an anti-bacterial peptide to treat methicillin-resistant *Staphylococcus aureus* (MRSA) (Liu et al., 2018). Immunomimetic designer cells were engineered through expression of human toll-like receptors (TLRs), triggering the NF-κB pathway upon activation. This endogenous pathway was re-routed to a synthetic expression unit to express lysostaphin, a bacteriolytic enzyme highly lethal to *Staphylococcus aureus.*

**Table 1:** Examples of closed-loop systems implemented in artificial designer cells

Ligand	Sensor platform	Processor platform	Therapeutic response	Disease	References
Dopamine	Human dopamine receptor 1 (DRD1)	Endogenous pathway (cAMP)	Atrial natriuretic peptide (ANP)	High blood pressure	Rössger et al. (2013b)
Bile acid	TGR5	Endogenous pathway (cAMP)	Hepatocyte growth factor (HGF)	Liver injury	Bai et al. (2016)
Formyl peptides	FPR1	Endogenous pathway (Calcium)	Autoinducer-2 (AI-2)	Anti-infection therapy	Sedlmayer et al. (2018)
TNF and IL22	TNFR and IL22R	NFκB and JAK/STAT3 pathways	IL4, IL10	Psoriasis	Schukur et al. (2015)
[Fatty acid] ↑	PPARa/TtgR	Orthogonal/endogenous pathways	Pramlintide	Obesity	Rössger et al. (2013a)
Thyroid hormones	TRa/Gal4 (TSR)	Coactivator/corepressor endogenous proteins	Thyroid hormone stimulating receptor antagonists	Grave’s disease	Saxena et al. (2016a)
[Glucose] ↑	Cav1.2 ion channel	Endogenous pathway (Calcium)	Insulin hGLP1	Type 1 and 2 diabetes	Xie et al. (2016b)
[H^+^] ↑	TDAG8	Endogenous pathway (cAMP)	Insulin	Diabetes	Ausländer et al. (2014)
Uric acid	KRAB-HucR (mUTS)	orthogonal	Urate oxidase	Hyperuricemia	Kemmer et al. (2010)
[Glucose] ↑	Glucose oxidase, catalase, gramicidin A	Synthetic vesicles containing insulin and sensitive to [H^+^]	Insulin release	Diabetes	Chen et al. (2018)

Although designer cells are often built to treat various diseases, there are some examples where closed-loop genetic circuits in designer cells have also been used for early diagnosis. In this case, the sensor system in the engineered cell is rewired to generate an alert signal rather than to produce a therapeutic protein. For example, a “biomedical tattoo” was designed, where cells engineered to sense hypercalcemia, an early marker often associated with cancer, were altered to produce black melanin (Tastanova et al., 2018). To develop this designer cell-based biomedical tattoo, a calcium-sensing receptor (CaSR) was rewired to a synthetic expression unit that produces tyrosinase upon activation. In the native context, tyrosinase is produced in specialized pigment-producing organelles (melanosomes) in melanocytes, where it catalyzes the oxidation of phenols such as tyrosine to form melanin, a black pigment, to protect the body from harmful UV radiation. In non-melanogenic designer cells, however, tyrosinase produces melanin in response to persistently increased blood Ca^2+^ levels.

## OPEN-LOOP GENE NETWORK SYSTEMS

**Figure 3:**
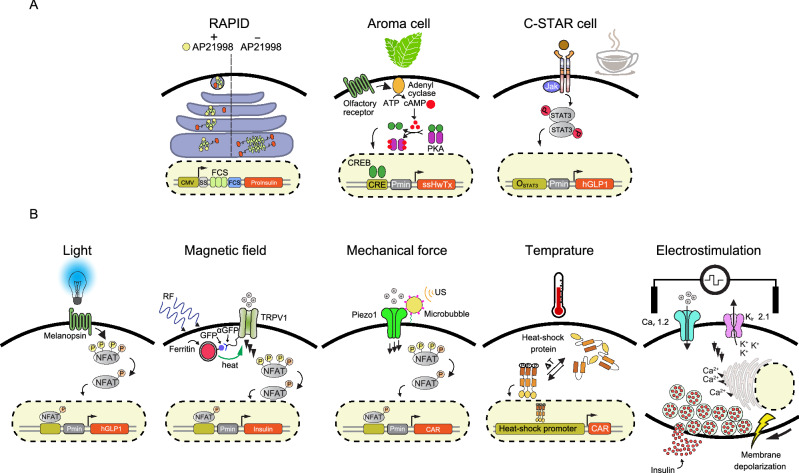
**Open-loop mediated cell-based therapies.** (A) Control of cell behaviors by chemical molecules. The RAPID cell is engineered to release a pre-formed protein from the ER upon addition of AP22542. A construct encoding a therapeutic gene (insulin or growth factor) is fused to a modified FKBP and spaced apart with a furin site. This construct aggregates in ER, but can monomerize in the presence of AP22542 and is trafficked to the plasma membrane, and releases the cleaved therapeutic protein with fast kinetics. The Aroma cell expresses an olfactory receptor that senses volatile spearmint and produces huwentoxin-IV by activating an endogenous cAMP pathway. C-STAR was developed based on dimerization of a chimeric receptor upon coffee administration. The activated receptor harnesses a JAK/STAT3 pathway to express hGLP1 in type-2 diabetes. (B) Physical cues to program designer cells. The opto-HEK cell contains a melanopsin receptor that allows calcium influx upon blue-light illumination, leading to expression of hGLP1. A magneto-thermal strategy was used, applying magnetic nanoparticles to stimulate TRPV1 channels. This system triggers calcium influx into HEK cells, leading to expression of insulin upon stimulation at radio-wave frequency (465 kHz, 23–32 mT). Ectopic expression of the mechanosensitive Piezo1 ion channel on T cells could also triggers calcium influx and related downstream transcription factors (e.g., NFAT) upon low-frequency ultrasound stimulation. This system was implemented in order to remotely and precisely induce expression of anti-CD19 chimeric antigen receptors (CARs) on T cells at tumor sites. Hyperthermal stimulation of T cells equipped with a synthetic expression unit containing a binding site for heat-shock proteins allowed them to wirelessly induce expression of CAR at a desired tumor site. Electrostimulation of _Electro_β cells engineered to express voltage-gated calcium channel (Ca_V_1.2) coupled to an inwardly rectifying potassium channel (K_ir_2.1) enabled rapid release of pre-formed insulin (within 20 min upon electrostimulation)

Open-loop systems do not have any genetically encoded negative feedback loop, but often use exogeneous inducers (chemical compounds and physical cues) instead in order to activate gene expression (Xie et al., 2016a). Chemical inducer molecules include organic and inorganic compounds, stimulatory peptides and volatile odorants (Fig. 3A). For example, Rivera and colleagues have engineered mammalian cells to enable rapid secretion of insulin after administration of AP22542, a chemical compound that can bind the FK506 binding protein (FKBP12) (Rivera et al., 2000). This strategy relies on expression of a construct encoding a regulatory domain containing multiple repeats of FKBP, connected to insulin by a linker containing a furin cleavage site. In the absence of AP22542, the FKBP-insulin fusion protein aggregates in the Endoplasmic Reticulum (ER), blocking efficient trafficking towards the Golgi apparatus. Addition of AP22542, in turn, causes FKBP to dissociate, releasing the fusion protein from the ER and allowing transport to the Golgi. In the trans-Golgi, a furin protease cleaves off the insulin moiety, which can then be functionally secreted. In their study, Rivera and colleagues showed that delivery of insulin is possible within 2 h after induction with AP22542, which was sufficient to attenuate the disease symptoms in experimental type-1 diabetes. In addition to inorganic chemical compounds, edible molecules from our daily diet can also be used for induction of designer cells. For example, C-STAR (caffeine-stimulated advanced regulators) designer cells are engineered cells that can produce GLP1, a clinically licensed therapeutic protein for type-2 diabetes, upon drinking commercially available coffee (Bojar et al., 2018). C-STAR cells are based on caffeine-induced dimerization and subsequent activation of chimeric receptors that trigger the JAK-STAT3 pathway. This genetic circuit was rewired to a synthetic expression unit containing binding sites for STAT3 to drive expression of GLP1. This study showed that administration of coffee in type-2 diabetic mice implanted with C-STAR cells could improve glucose levels as well as other associated symptoms of diabetes in the mice. Odorants are another chemical-based stimulus used to program therapeutic mammalian designer cells. This was demonstrated by engineering designer “Aroma cells” to treat pain in mice (Wang et al., 2018a). Aroma cells were engineered to express a spearmint flavor-triggered olfactory receptor (G_olf_), which enabled them to activate the cAMP pathway upon administration of a volatile spearmint flavor. Elevated levels of cAMP were also rewired to transcription of a therapeutic protein called huwentoxin-IV, which selectively inhibits the pain-mediating voltage-gated sodium channel Na_V_1.7. Despite high induction potential and ease of use, potential clinical applications of chemical inducers are often limited by side effects, bioavailability or pharmacodynamics (Xie and Fussenegger, 2018). Chemical inducers can also diffuse freely and may cause side effects due to off-target activity elsewhere in the body. Additionally, it can be hard to remove the inducer quickly and reliably if required (Beyer et al., 2015; Müller et al., 2015).

Conversely, traceless physical inducers can provide a robust, efficient and precise way to wirelessly control cellular behaviors at a desired time and place. Approaches based on physical inducers also reduce the likelihood of cross-reactivity and off-target effects, and additionally avoid the need for invasive access to the tissue or targeted organ. A toolbox of traceless inducers, including light, magnetic and mechanical forces, heat and electrical stimulation, is available for non-invasive, safe, and reliable physical stimulation to remotely control engineered cells (Fig. 3B). Here we summarize some of the most important studies that have implemented physical inducers to program therapeutic mammalian cells. A list of remote-controllable designer cells and their applications in translational medicine is provided in Table 2. In optogenetics, light serves as an input signal to stimulate a genetic circuit in designer cells (Kolar and Weber, 2017). Light has unique features, such as high spatiotemporal resolution as well as excellent tunability and ease of reversibility to control cellular behavior (Miller et al., 2018). An optogenetically engineered artificial mammalian cell was developed by introducing a genetic circuit containing a light-sensitive receptor and a synthetic promoter driving expression of a therapeutic protein in HEK cells. These opto-HEK cells were engineered to express melanopsin, a blue-light-sensitive Gq-linked GPCR, which triggers the calcium pathway leading to production of GLP1 from a synthetic expression cassette (Ye et al., 2011). It was shown that blue-light illumination improved blood glucose hemostasis in an experimental mouse model of type-2 diabetes implanted with opto-HEK cells. Mechanical perturbation is another traceless physical approach to program designer cells through the introduction of mechanosensitive ion channels or receptors (Zhu et al., 2020). Such a mechanogenetics strategy can include acoustically mediated shear stress generated through compression and stretching (Dufort et al., 2011). For example, expression of the mechanosensitive ion channel Piezo1 triggered calcium influx in T cells upon low-frequency ultrasound stimulation, triggering expression of anti-CD19 chimeric antigen receptors (CARs) in tumor sites remotely and with precise timing (Pan et al., 2018). Magnetogenetics also provides a facile wireless approach to remotely program designer cells based on externally applied magnetic fields (Christiansen et al., 2020). Stanley and colleagues have developed a magneto-thermo strategy using activated magnetic nanoparticles to stimulate a thermosensitive ion channel (TRPV1) to trigger calcium influx (Stanley et al., 2012). In their work, a rise in local temperature produced by magnetic fields at radio-wave frequency (465 kHz, 23–32 mT) was used to produce and release insulin from a synthetic calcium-responsive expression unit in engineered HEK cells. In thermogenetics, a change in temperature can be used to stimulate designer cells. So far, engineered cells have been designed that are directly controlled by either low temperature (cooling, hypothermia) or high temperature (heating, hyperthermia) (Miller et al., 2018; Bai et al., 2019). For instance, raising the temperature normally induces cellular stress, resulting in activation of related transcription factors, such as heat-shock proteins. Using a synthetic thermal promoter based on the heat-shock protein 70B′ promoter (HSPA6 promoter), engineered T cells could be non-invasively induced at the tumor site using heat (Miller et al., 2018). Electrogenetics is another traceless technique that employs electrical pulses to directly program the behavior of designer cells. More recently, an electro designer cell was developed through ectopic expression of a voltage-gated circuit including a voltage-gated calcium channel (Ca_V_1.2) coupled to an inwardly rectifying potassium channel (K_ir_2.1) (Krawczyk et al., 2020). Implementing this genetic circuit could mediate an influx of calcium into the cell upon electrostimulation, and the resulting elevated cytosolic calcium concentration triggered expression of a transgene from a synthetic expression unit. In order to develop an engineered cell with faster kinetics, a similar voltage-gated circuit was introduced into a pancreatic β cell line, termed _Electro_β cells, to release pre-formed insulin within 20 min upon electrostimulation. Stimulated _Electro_β cells encased in a subcutaneously implanted electronic device rapidly reversed hyperglycemia in an alloxan-induced type-1 diabetic mouse model.

**Table 2:** Examples of remote-controllable designer cells

Inducer	Sensor platform	Processor platform	Therapeutic response	Disease	References
Light	Human melanopsin	Calcium pathway	GLP1	T2D*	Ye et al. (2011)
	Opto-CRAC	Calcium pathway	IL12 and TNFα expression	Cancer	He et al. (2015)
	LightOn	Orthogonal pathway	Insulin	T1D**	Wang et al. (2012)
	FRL-v2	Endogenous/orthogonal	GLP1	T2D	Shao et al. (2017)
Magnetic field	Thermo-sensitive channels (TRPV1)	Calcium pathway	Insulin	T1D	Stanley et al. (2012)
Mechanical disturbance	Mechanosensitive ion channels (Piezo1)	Calcium pathway	CAR	Cancer	Pan et al. (2018)
Temperature	TRMP8 ion channel	Calcium Pathway	Insulin, mActRIIBECD-hFc***	T1D, Muscle atrophy	Bai et al. (2019)
Electrical stimulation	Voltage gated channels (Cav1.2/Kir2.1)	Calcium pathway	Insulin secretion	T1D	Krawczyk et al. (2020)

*T2D; type-2 diabetes

**T1D; type-1 diabetes

***mActRIIBECD-hFc; modified, activin type IIB, receptor ligand trap protein

## CONCLUSIONS AND PERSPECTIVE

Disease targeting with engineered designer cells to produce on-demand therapeutic agents is moving beyond proof-of-concept, and cancer treatment with CAR-T cells is the pioneering application (Yip and Webster, 2018). Emerging new technologies in molecular biology, materials sciences and digital devices, especially in the fields of designer cell development, manufacturing and implantation, are expected to rapidly advance cell-based therapy in the future by delivering increased safety, efficiency, and specificity. Strategies at the molecular biology level are being developed to engineer cells for delivery of biological cargos to mammalian cells with maximum efficiency and minimum cytotoxicity along with reduced associated risks (Marx, 2015). Although there are relatively reliable methods to deliver DNA, delivery of large constructs (e.g., multiple genetic circuit) to cells is still challenging (Mansouri et al., 2016). In addition, integration of genetic circuits into the genome of the host is another process that needs careful control. The fast-growing field of genome editing and its applications that use programmable nucleases, such as CRISPR/Cas9, have made it possible to integrate desired components into the genome of individual cells with high precision (Maggio and Gonçalves, 2015). For example, synthetic gene circuits can be integrated at a safe-harbor locus (e.g., AAVS1) to prevent transgene silencing and to ensure robust long-term performance (Papapetrou and Schambach, 2016). Manufacturing of engineered cells on a sufficient scale for clinical requirements is another challenge in cell-based therapy. In general, scale-up is easier for artificial cells, which are more compatible with bioreactors, than for primary cells or stem cells. Activation of immune system responses upon cell transplantation in patients is also a major concern. Human-derived genetic circuits can reduce possible immunogenic responses upon cell implantation (Israni et al., 2021). Therefore, increased use of humanized genetic circuits in engineered cells can potentially minimize the risk of immune system responses. Safety switches that allow the user to turn the synthetic genetic network “OFF” are crucial for any clinical application. In addition, genetically modified patient-derived cells can be used to reduce possible host immune responses. Cell encapsulation is another strategy to enable implanted designer cells to evade immune system responses. New cell encapsulation strategies that ensure long-term transplantation of designer cells need to be developed through materials engineering (Desai and Shea, 2017; Bose et al., 2020). Furthermore, miniaturized biocompatible materials for encasing designer cells are of special interest for optogenetics- and electrogenetics-mediated cell therapies. Incorporation of digital technologies such as wearable electronic devices can also foster personalized cell-based therapy (Kim et al., 2019). Wearable smart devices such as smartphones and smart watches are either equipped with health-related biosensor systems or can be wirelessly connected to specialized medical sensor devices. Therefore, smart devices provide a reliable sensor platform to serve as a receiver unit that senses the biomarker level and transfers the data to the patient (Sim, 2019), enabling the patient to activate designer cells through chemical compounds or physical cues (e.g., light) in order to regulate the level of therapeutics to be administered (Mansouri et al., 2021b, a). In addition, digital devices can potentially collect daily health-related data and analyze them through installed artificial algorithms or machine-learning models. The processed physiological information can be integrated with other health-related data through the internet of things (IOT) and finally transferred to a clinician for continuous monitoring of a patient’s status (Steinhubl et al., 2015). Furthermore, programming of designer cells for on-demand release of therapeutics at the right time would allow the physician to control the therapy remotely (Sim, 2019).

In this review, we have introduced the principal design parameters for genetic circuits in mammalian cells, in order to enable the cells to sense a user-defined ligand, process it, and respond in a customized way. Challenges remain in engineering of cells for therapeutic purposes, but the field of synthetic biology is rapidly progressing and further improvements in applications for early detection and therapeutic interventions can be expected in the near future.

## References

[CR1] Albinger N, Hartmann J, Ullrich E (2021). Current status and perspective of CAR-T and CAR-NK cell therapy trials in Germany. Gene Ther.

[CR2] Ausländer D, Ausländer S, Charpin-El Hamri G, Sedlmayer F, Müller M, Frey O, Hierlemann A, Stelling J, Fussenegger M (2014). A synthetic multifunctional mammalian pH sensor and CO_2_ transgene-control device. Mol Cell.

[CR3] Bai P, Ye H, Xie M, Saxena P, Zulewski H, Charpin-El Hamri G, Djonov V, Fussenegger M (2016). A synthetic biology-based device prevents liver injury in mice. J Hepatol.

[CR4] Bai P, Liu Y, Xue S, Hamri GCE, Saxena P, Ye H, Xie M, Fussenegger M (2019). A fully human transgene switch to regulate therapeutic protein production by cooling sensation. Nat Med.

[CR5] Beyer HM, Naumann S, Weber W, Radziwill G (2015). Optogenetic control of signaling in mammalian cells. Biotechnol J.

[CR6] Bojar D, Scheller L, Hamri GCE, Xie M, Fussenegger M (2018). Caffeine-inducible gene switches controlling experimental diabetes. Nat Commun.

[CR7] Bose S, Volpatti LR, Thiono D, Yesilyurt V, McGladrigan C, Tang Y, Facklam A, Wang A, Jhunjhunwala S, Veiseh O (2020). A retrievable implant for the long-term encapsulation and survival of therapeutic xenogeneic cells. Nat Biomed Eng.

[CR8] Brown SA, Kovatchev BP, Raghinaru D, Lum JW, Buckingham BA, Kudva YC, Laffel LM, Levy CJ, Pinsker JE, Wadwa RP (2019). Six-month randomized, multicenter trial of closed-loop control in type 1 diabetes. N Engl J Med.

[CR9] Caliendo F, Dukhinova M, Siciliano V (2019). Engineered cell-based therapeutics: synthetic biology meets immunology. Front Bioeng Biotechnol.

[CR10] Carpenter A, Paulsen I, Williams T (2018). Blueprints for biosensors: design, limitations, and applications. Genes (basel).

[CR11] Chen Z, Wang J, Sun W, Archibong E, Kahkoska AR, Zhang X, Lu Y, Ligler FS, Buse JB, Gu Z (2018). Synthetic beta cells for fusion-mediated dynamic insulin secretion. Nat Chem Biol.

[CR12] Chmielewski M, Abken H (2015). TRUCKs: the fourth generation of CARs. Expert Opin Biol Ther.

[CR13] Cho JH, Collins JJ, Wong WW (2018). Universal chimeric antigen receptors for multiplexed and logical control of T cell responses. Cell.

[CR14] Choi BD, Yu X, Castano AP, Bouffard AA, Schmidts A, Larson RC, Bailey SR, Boroughs AC, Frigault MJ, Leick MB (2019). CAR-T cells secreting BiTEs circumvent antigen escape without detectable toxicity. Nat Biotechnol.

[CR15] Christiansen MG, Hornslien W, Schuerle PS, Schuerle S (2020). A possible inductive mechanism for magnetogenetics. bioRxiv.

[CR16] Desai T, Shea LD (2017). Advances in islet encapsulation technologies. Nat Rev Drug Discov.

[CR17] Dixon TA, Williams TC, Pretorius IS (2021). Sensing the future of bio-informational engineering. Nat Commun.

[CR18] Dufort CC, Paszek MJ, Weaver VM (2011). Balancing forces: architectural control of mechanotransduction. Nat Rev Mol Cell Biol.

[CR19] Eglen R, Reisine T (2011). Primary cells and stem cells in drug discovery: emerging tools for high-throughput screening. Assay Drug Dev Technol.

[CR20] Fedorov VD, Themeli M, Sadelain M (2013). PD-1- and CTLA-4-based inhibitory chimeric antigen receptors (iCARs) divert off-target immunotherapy responses. Sci Transl Med.

[CR21] Gaj T, Sirk SJ, Shui S-L, Liu J (2016). Genome-editing technologies: principles and applications. Cold Spring Harb Perspect Biol.

[CR22] He L, Zhang Y, Ma G, Tan P, Li Z, Zang S, Wu X, Jing J, Fang S, Zhou L (2015). Near-infrared photoactivatable control of Ca2+ signaling and optogenetic immunomodulation. Elife.

[CR23] Israni DV, Li H-S, Gagnon KA, Sander JD, Roybal KT, Joung JK, Wong WW, Khalil AS (2021). Clinically-driven design of synthetic gene regulatory programs in human cells. bioRxiv.

[CR24] June CH, Sadelain M (2018). Chimeric antigen receptor therapy. N Engl J Med.

[CR25] Kemmer C, Gitzinger M, Daoud-El Baba M, Djonov V, Stelling J, Fussenegger M (2010). Self-sufficient control of urate homeostasis in mice by a synthetic circuit. Nat Biotechnol.

[CR26] Khalil AS, Collins JJ (2010). Synthetic biology: applications come of age. Nat Rev Genet.

[CR27] Kiel C, Yus E, Serrano L (2010). Engineering signal transduction pathways. Cell.

[CR28] Kim J, Campbell AS, de Ávila BEF, Wang J (2019). Wearable biosensors for healthcare monitoring. Nat Biotechnol.

[CR29] Kitada T, DiAndreth B, Teague B, Weiss R (2018). Programming gene and engineered-cell therapies with synthetic biology. Science.

[CR30] Klichinsky M, Ruella M, Shestova O, Lu XM, Best A, Zeeman M, Schmierer M, Gabrusiewicz K, Anderson NR, Petty NE (2020). Human chimeric antigen receptor macrophages for cancer immunotherapy. Nat Biotechnol.

[CR31] Kloss CC, Condomines M, Cartellieri M, Bachmann M, Sadelain M (2013). Combinatorial antigen recognition with balanced signaling promotes selective tumor eradication by engineered T cells. Nat Biotechnol.

[CR32] Kojima R, Aubel D, Fussenegger M (2016). Toward a world of theranostic medication: programming biological sentinel systems for therapeutic intervention. Adv Drug Deliv Rev.

[CR33] Kojima R, Scheller L, Fussenegger M (2018). Nonimmune cells equipped with T-cell-receptor-like signaling for cancer cell ablation. Nat Chem Biol.

[CR34] Kojima R, Aubel D, Fussenegger M (2020). Building sophisticated sensors of extracellular cues that enable mammalian cells to work as “doctors” in the body. Cell Mol Life Sci.

[CR35] Kolar K, Weber W (2017). Synthetic biological approaches to optogenetically control cell signaling. Curr Opin Biotechnol.

[CR36] Krawczyk K, Xue S, Buchmann P, Charpin-El-Hamri G, Saxena P, Hussherr MD, Shao J, Ye H, Xie M, Fussenegger M (2020). Electrogenetic cellular insulin release for real-time glycemic control in type 1 diabetic mice. Science.

[CR37] Li J, Liang JY, Laken SJ, Langer R, Traverso G (2020). Clinical opportunities for continuous biosensing and closed-loop therapies. Trends Chem.

[CR38] Lim WA, June CH (2017). Review the principles of engineering immune cells to treat cancer. Cell.

[CR39] Liu Y, Bai P, Woischnig AK, Charpin-El Hamri G, Ye H, Folcher M, Xie M, Khanna N, Fussenegger M (2018). Immunomimetic designer cells protect mice from MRSA infection. Cell.

[CR40] Lohmueller J, Butchy A, Tivon Y, Kvorjak M, Miskov-Zivanov N, Deiters A, Finn O (2020). Post-translational covalent assembly of CAR and synNotch receptors for programmable antigen targeting. bioRxiv.

[CR41] Maggio I, Gonçalves MAFV (2015). Genome editing at the crossroads of delivery, specificity, and fidelity. Trends Biotechnol.

[CR42] Mansouri M, Berger P (2018). Multigene delivery in mammalian cells: recent advances and applications. Biotechnol Adv.

[CR43] Mansouri M, Bellon-Echeverria I, Rizk A, Ehsaei Z, Cianciolo Cosentino C, Silva CS, Xie Y, Boyce FM, Davis MW (2016). Highly efficient baculovirus-mediated multigene delivery in primary cells. Nat Commun.

[CR44] Mansouri M, Strittmatter T, Fussenegger M (2019). Light-controlled mammalian cells and their therapeutic applications in synthetic biology. Adv Sci (weinh)..

[CR45] Mansouri M, Hussherr M-D, Strittmatter T, Buchmann P, Xue S, Camenisch G, Fussenegger M (2021). Smart-watch-programmed green-light-operated percutaneous control of therapeutic transgenes. Nat Commun.

[CR46] Mansouri M, Xue S, Hussherr M-D, Strittmatter T, Camenisch G, Fussenegger M (2021). Smartphone-flashlight-mediated remote control of rapid insulin secretion restores glucose homeostasis in experimental type-1 diabetes. Small.

[CR47] Marx V (2015). Cell biology: delivering tough cargo into cells. Nat Methods.

[CR48] Matsunaga S, Jeremiah SS, Miyakawa K, Kurotaki D, Shizukuishi S, Watashi K, Nishitsuji H, Kimura H, Tamura T, Yamamoto N (2020). Engineering cellular biosensors with customizable antiviral responses targeting hepatitis B virus. Science.

[CR49] Maurisse R, De Semir D, Emamekhoo H, Bedayat B, Abdolmohammadi A, Parsi H, Gruenert DC (2010). Comparative transfection of DNA into primary and transformed mammalian cells from different lineages. BMC Biotechnol.

[CR50] McClune CJ, Alvarez-Buylla A, Voigt CA, Laub MT (2019). Engineering orthogonal signalling pathways reveals the sparse occupancy of sequence space. Nature.

[CR51] Miller IC, Gamboa Castro M, Maenza J, Weis JP, Kwong GA (2018). Remote control of mammalian cells with heat-triggered gene switches and photothermal pulse trains. ACS Synth Biol.

[CR52] Morsut L, Roybal KTKT, Xiong X, Gordley RM, Coyle SM, Thomson M, Lim WAWA (2016). Engineering customized cell sensing and response behaviors using synthetic notch receptors. Cell.

[CR53] Mukherjee A, Repina NA, Schaffer DV, Kane RS (2017). Optogenetic tools for cell biological applications. J Thorac Dis.

[CR54] Müller K, Naumann S, Weber W, Zurbriggen MD (2015). Optogenetics for gene expression in mammalian cells. Biol Chem.

[CR55] Mure LSS, Hatori M, Zhu Q, Demas J, Kim IMM, Nayak SKK, Panda S (2016). Melanopsin-encoded response properties of intrinsically photosensitive retinal ganglion cells. Neuron.

[CR56] Neelapu SS, Tummala S, Kebriaei P, Wierda W, Gutierrez C, Locke FL, Komanduri KV, Lin Y, Jain N, Daver N (2018). Chimeric antigen receptor T-cell therapy-assessment and management of toxicities. Nat Rev Clin Oncol.

[CR57] Pan Y, Yoon S, Sun J, Huang Z, Lee C, Allen M, Wu Y, Chang YJ, Sadelain M, Kirk Shung K (2018). Mechanogenetics for the remote and noninvasive control of cancer immunotherapy. Proc Natl Acad Sci USA.

[CR58] Papapetrou EP, Schambach A (2016). Gene insertion into genomic safe harbors for human gene therapy. Mol Ther.

[CR59] Park JS, Rhau B, Hermann A, McNally KA, Zhou C, Gong D, Weiner OD, Conklin BR, Onuffer J, Lim WA (2014). Synthetic control of mammalian-cell motility by engineering chemotaxis to an orthogonal bioinert chemical signal. Proc Natl Acad Sci USA.

[CR60] Polonsky KS, Given BD, Van Cauter E (1988). Twenty-four-hour profiles and pulsatile patterns of insulin secretion in normal and obese subjects. J Clin Invest.

[CR61] Rafiq S, Hackett CS, Brentjens RJ (2020). Engineering strategies to overcome the current roadblocks in CAR T cell therapy. Nat Rev Clin Oncol.

[CR62] Rivera VM, Wang X, Wardwell S, Courage NL, Volchuk A, Keenan T, Holt DA, Gilman M, Orci L, Cerasoli F (2000). Regulation of protein secretion through controlled aggregation in the endoplasmic reticulum. Science.

[CR63] Rössger K, Charpin-El-Hamri G, Fussenegger M (2013). A closed-loop synthetic gene circuit for the treatment of diet-induced obesity in mice. Nat Commun.

[CR64] Rössger K, Charpin-El Hamri G, Fussenegger M (2013). Reward-based hypertension control by a synthetic brain-dopamine interface. Proc Natl Acad Sci USA.

[CR65] Roybal KT, Lim WA (2017). Synthetic immunology: hacking immune cells to expand their therapeutic capabilities. Annu Rev Immunol.

[CR66] Roybal KT, Rupp LJ, Morsut L, Walker WJ, McNally KA, Park JS, Lim WA, Box C, Eccles SA, Maher J (2016). Precision tumor recognition by T cells with combinatorial antigen-sensing circuits. Cell.

[CR67] Roybal KT, Williams JZ, Morsut L, Rupp LJ, Kolinko I, Choe JH, Walker WJ, McNally KA, Lim WA, Rupp LJ (2016). Engineering T cells with customized therapeutic response programs using synthetic notch receptors. Cell.

[CR68] Saxena P, Hamri GCE, Folcher M, Zulewski H, Fussenegger M (2016). Synthetic gene network restoring endogenous pituitary-thyroid feedback control in experimental Graves’ disease. Proc Natl Acad Sci USA.

[CR69] Saxena P, Heng BC, Bai P, Folcher M, Zulewski H, Fussenegger M (2016). A programmable synthetic lineage-control network that differentiates human IPSCs into glucose-sensitive insulin-secreting beta-like cells. Nat Commun.

[CR70] Saxena P, Bojar D, Zulewski H, Fussenegger M (2017). Generation of glucose-sensitive insulin-secreting beta-like cells from human embryonic stem cells by incorporating a synthetic lineage-control network. J Biotechnol.

[CR71] Scheller L, Fussenegger M (2019). From synthetic biology to human therapy: engineered mammalian cells. Curr Opin Biotechnol.

[CR72] Scheller L, Strittmatter T, Fuchs D, Bojar D, Fussenegger M (2018). Generalized extracellular molecule sensor platform for programming cellular behavior. Nat Chem Biol.

[CR73] Schmidt P, Raftery MJ, Pecher G (2021). Engineering NK cells for CAR therapy—recent advances in gene transfer methodology. Front Immunol.

[CR74] Schukur L, Fussenegger M (2016). Engineering of synthetic gene circuits for (re-)balancing physiological processes in chronic diseases. Wiley Interdiscip Rev Syst Biol Med.

[CR75] Schukur L, Geering B, Charpin-El Hamri G, Fussenegger M (2015). Implantable synthetic cytokine converter cells with AND-gate logic treat experimental psoriasis. Sci Transl Med.

[CR76] Sedlmayer F, Hell D, Müller M, Ausländer D, Fussenegger M (2018). Designer cells programming quorum-sensing interference with microbes. Nat Commun.

[CR77] Shao J, Xue S, Yu G, Yu Y, Yang X, Bai Y, Zhu S, Yang L, Yin J, Wang Y (2017). Smartphone-controlled optogenetically engineered cells enable semiautomatic glucose homeostasis in diabetic mice. Sci Transl Med.

[CR78] Sim I (2019). Mobile devices and health. N Engl J Med.

[CR79] Slattery D, Choudhary P (2017). Clinical use of continuous glucose monitoring in adults with type 1 diabetes. Diabetes Technol Ther.

[CR80] Stanley SA, Gagner JE, Damanpour S, Yoshida M, Dordick JS, Friedman JM (2012). Radio-wave heating of iron oxide nanoparticles can regulate plasma glucose in mice. Science.

[CR81] Steinhubl SR, Muse ED, Topol EJ (2015). The emerging field of mobile health. Sci. Transl. Med..

[CR82] Su Y, Huang X, Huang Z, Huang T, Li T, Fan H, Zhang K, Yi C (2020). Early but not delayed optogenetic RAF activation promotes astrocytogenesis in mouse neural progenitors. J Mol Biol.

[CR83] Sun J, Lei L, Tsai CM, Wang Yi, Shi Y, Ouyang M, Lu S, Seong J, Kim TJ, Wang P (2017). Engineered proteins with sensing and activating modules for automated reprogramming of cellular functions. Nat Commun.

[CR84] Tastanova A, Folcher M, Müller M, Camenisch G, Ponti A, Horn T, Tikhomirova MS, Fussenegger M (2018). Synthetic biology-based cellular biomedical tattoo for detection of hypercalcemia associated with cancer. Sci Transl Med.

[CR85] Toda S, Blauch LR, Tang SKY, Morsut L, Lim WA (2018). Programming self-organizing multicellular structures with synthetic cell-cell signaling. Science.

[CR86] Wang X, Chen X, Yang Y (2012). Spatiotemporal control of gene expression by a light-switchable transgene system. Nat Methods.

[CR87] Wang H, Xie M, Charpin-El Hamri G, Ye H, Fussenegger M (2018). Treatment of chronic pain by designer cells controlled by spearmint aromatherapy. Nat Biomed Eng.

[CR88] Wang Y, Wang M, Dong K, Ye H (2018). Engineering mammalian designer cells for the treatment of metabolic diseases. Biotechnol J.

[CR89] Weber W, Fussenegger M (2004). Inducible gene expression in mammalian cells and mice. Methods Mol Biol.

[CR90] Xie M, Fussenegger M (2015). Mammalian designer cells: engineering principles and biomedical applications. Biotechnol J.

[CR91] Xie M, Fussenegger M (2018). Designing cell function: assembly of synthetic gene circuits for cell biology applications. Nat Rev Mol Cell Biol.

[CR92] Xie M, Haellman V, Fussenegger M (2016). Synthetic biology - application-oriented cell engineering. Curr Opin Biotechnol.

[CR93] Xie M, Ye H, Wang H, Charpin-El Hamri G, Lormeau C, Saxena P, Stelling J, Fussenegger M (2016). β-cell-mimetic designer cells provide closed-loop glycemic control. Science.

[CR94] Xue S, Yin J, Shao J, Yu Y, Yang L, Wang Y, Xie M, Fussenegger M, Ye H (2017). A synthetic-biology-inspired therapeutic strategy for targeting and treating hepatogenous diabetes. Mol Ther.

[CR95] Yamada M, Nagasaki SC, Ozawa T, Imayoshi I (2020). Light-mediated control of gene expression in mammalian cells. Neurosci Res.

[CR96] Ye H, Baba MDE, Peng RW, Fussenegger M (2011). A synthetic optogenetic transcription device enhances blood-glucose homeostasis in mice. Science.

[CR97] Yip A, Webster RM (2018). The market for chimeric antigen receptor T cell therapies. Nat Rev Drug Discov.

[CR98] Zhang F, Tzanakakis ES (2019). Amelioration of diabetes in a murine model upon transplantation of pancreatic β-cells with optogenetic control of cyclic adenosine monophosphate. ACS Synth Biol.

[CR99] Zhu L, Wu Y, Yoon CW, Wang Y (2020). Mechanogenetics for cellular engineering and cancer immunotherapy. Curr Opin Biotechnol.

